# Enhancing surgical safety through surgical instruments repair technicians’ training: recent experience from Nigeria

**DOI:** 10.3389/fpubh.2025.1522315

**Published:** 2025-02-18

**Authors:** Chisom R. Udeigwe-Okeke, Justina O. Seyi-Olajide, Aderonke O. Obisesan, Keith Miles, Nkeiruka Obi, Emmanuel A. Ameh

**Affiliations:** ^1^Department of Surgery, National Hospital, Abuja, Nigeria; ^2^NSOANP Technical Review Committee, Abuja, Nigeria; ^3^Department of Surgery, Lagos University Teaching Hospital, Lagos, Nigeria; ^4^Department of Anaesthesia, National Hospital, Abuja, Nigeria; ^5^Safe Surgery Initiative, Phoenix, AZ, United States; ^6^Smile Train, Africa Office, Lagos, Nigeria

**Keywords:** surgical safety, instrument repair, training program, biomedical technicians, operating room nurses

## Abstract

**Background:**

Faulty or poorly maintained surgical instruments increase risks of complications, prolong operating times, and reduce efficiency, especially in low- and middle-income countries (LMICs). To address this, Nigeria introduced the Surgical Instruments Repair Technicians (SIRT) program, to improve instrument safety.

**Objective:**

This study evaluated the SIRT program’s initial impact, sustainability, and scalability for improved surgical instrument maintenance in LMICs.

**Methods:**

The program was deployed in two phases. Phase one involved online theoretical and hands-on training for biomedical technicians and operating room/central sterile supply department nurses from Smile Train partner and public hospitals across Nigeria’s six geopolitical zones. Participants were provided repair kits to establish institutional workbenches. Phase two focused on expanding training with a one-week hands-on program. Data on demographics, training feedback, and repair outcomes were collected.

**Results:**

A total of 36 participants completed training (24 in phase one, 12 in phase two), evaluating 1,623 instruments with a 99.6% successful repair rate. Post-training surveys showed that 83.3% of participants felt more confident identifying faulty instruments, and 95.8% reported adequate repair skills. Institutional workbenches were established in 50% of hospitals, and repair drives were conducted within institutions and neighboring hospitals.

**Conclusion:**

The program demonstrated significant potential for improving surgical instrument maintenance and enhancing safety in LMICs. Integrating the program into hospital budgets could support sustainable expansion.

## Introduction

The World Health Assembly resolution 68.15 on strengthening emergency and essential surgical and anesthesia care as a component of universal health coverage laid the groundwork for initial and ongoing efforts to improve access to safe, timely and affordable surgical care, especially in low- and middle-income countries (1). In line with this mandate, Nigeria launched its National Surgical, Obstetrics, Anesthesia and Nursing Plan (NSOANP) in 2019 (2). This policy incorporated into its priorities an emphasis on surgical safety as a key aspect of scaling up access to surgical, obstetric and anesthesia care.

The World Health Organization (WHO), in recognition of the importance of safe surgery, has undertaken several global and regional initiatives on surgical safety (3). One of these is the WHO surgical safety checklist, which addresses the sterility of instruments as one of its objectives (4). In addition to sterility, however, proper instrument maintenance is extremely important. This is because surgical instruments are carefully designed and expertly crafted to perform specific tasks with the highest levels of accuracy (5). Maintaining precision and efficiency during surgical procedures reduces the risk of complications and improves outcomes (5). Using efficient instruments also shortens the operation time, which would otherwise be prolonged by faulty or ineffective instruments.

Up to 1,500 incidents of poor-quality surgical instruments causing harm may occur annually (6). This number may be greater for low- and middle-income countries (LMICs), as old and faulty instruments are repeatedly used for surgeries, and appropriate instrument care or repair expertise is limited. In addition, the cost of acquiring and replacing instruments poses a significant financial burden in these countries. Unfortunately, even newly purchased surgical instruments are not entirely safe because up to 15 to 35% of newly purchased instruments from reputable suppliers fail to meet quality control standards on appraisal (7).

While existing instruments can easily be refurbished at far lower costs, in Nigeria and the West African subregion, few surgical instrument repair trainings or centers exist, and individual training from international bodies has high costs (8).

As part of the prioritization of safety in the scale-up of access to quality surgical care in Nigeria, surgical instrument maintenance was incorporated as a key implementation goal of the NSOANP. This was accomplished through the deployment of the Safe Surgery Initiative® curriculum on Surgical Instruments Repair Technicians (SIRT) training (9). The program trains biomedical technicians and operating room and central sterile supply department nurses in the inventory, repair and maintenance of surgical instruments.

This report evaluates the initial effectiveness and impact of the SIRT program in improving the maintenance and safety of surgical instruments across hospitals in Nigeria while assessing its potential for scaling up and replication within the country and across other LMICs.

## Materials and methods

The NSOANP implementation committee partnered with the cleft charity Smile Train Inc. to train relevant hospital staff on surgical instrument inventory, repair and maintenance. The training was deployed and coordinated by the Safe Surgery Initiative®.

### Participant selection

Nigeria has 36 states and is divided into six geopolitical zones (Northwest, Northeast, North Central, Southwest, Southeast and South South zones). The participants were selected from each of the six geopolitical zones to ensure equitable spread across the country. Participants were selected by the administrative heads of the Smile Train partner hospitals. The selected participants were biomedical technicians, operating room nurses and central sterile supply department nurses from Smile Train cleft lip and palate partner hospitals as well as other public tertiary hospitals across the country. Two participants were selected from each hospital chosen. The biomedical technicians had a general knowledge about surgical instruments while the operating room and central sterile supply department nurses only had experience with cleaning and sterilization of surgical instruments. None of the participants had prior in depth experience with repair and maintenance of surgical instruments.

### Training curriculum

The training was performed via the Safe Surgery Initiative® curriculum (Table 1).

**Table 1:** Surgical instruments repair technicians training curriculum.

Week	Day	Specialty focus	Instrument focus	Specific content
Week 1	Monday		Introduction	Training overview Lab equipment setup Safety protocols equipment operation
	Tuesday	General surgery	Haemostats/Forceps/Skin Rakes/Skin Hooks	Inspection Ratchet testing Refurbishment testing Esthetic (Buffing/Polish/Rust Removal)
	Wednesday	General surgery	Needle Holders/General Scissors (Mayo)	Inspection Refurbishment testing Esthetic (Buffing/Polish/Rust Removal)
	Thursday	Plastic/Ear, Nose & Throat surgery	Tenotomy/SuperCuts/Metzenbaum Scissors	Inspection Refurbishment testing Esthetic (Buffing/Polish/Rust Removal)
	Friday	Plastic/Ear, Nose & Throat surgery	Tenotomy/SuperCuts/Metzenbaum Scissors	Inspection Refurbishment testing Esthetic (Buffing/Polish/Rust Removal)
	Saturday	Review	Repair overview	Individual training Reviewing repair techniques Additional training on specific surgical tools
Week 2	Monday	Plastic/Ear, Nose & Throat surgery	Rasps/Suction Tubes/Awls	Inspection refurbishment Testing Esthetic (Buffing/Polish/Rust Esthetic)
	Tuesday	Dental/Oral surgery	Extractors/Pliers/Picks	Inspection Refurbishment testing Esthetic (Buffing/Polish/Rust Esthetic)
	Wednesday	Orthopedic surgery	Elevators/Gouges/Curettes	Inspection Refurbishment testing Esthetic (Buffing/Polish/Rust Esthetic)
	Thursday	Orthopaedic surgery	Osteotomes/Chisels/Amputation Knife	Inspection Refurbishment testing Esthetic (Buffing/Polish/Rust Esthetic)
	Friday	Neuro/Arthroscopic Surgery	Ronguers/Kerrison’s/Pituitary Graspers	Inspection Refurbishment testing Esthetic (Buffing/Polish/Rust Esthetic)
	Saturday	Continuing education	Repair overview	Individual training Reviewing repair techniques Additional training on specific surgical tools Certification discussion Provide participants with individualized continued learning plans to improve their surgical instrument refurbishment skills

### Training program

The training was deployed in two phases.

#### Phase one

The goals of this phase were to ensure the acquisition of the requisite skills for surgical instrument inventory, repair and maintenance and to create a pool of trainers for stepping down and expanding training across the country. This phase had 2 stages:

Stage 1: A 2-week online theoretical training module was completed 4 weeks prior to the second component of this phase. A participant was required to score a minimum of 80% in the post-training evaluation to qualify for stage 2.

Stage 2: A 2-week physical hands-on component was conducted by four previously trained local facilitators and one instructor from the Safe Surgery Initiative®. For this component, participants were encouraged to bring in faulty surgical instruments for repair in addition to what was provided for the training.

At the end of the training, each participating hospital received a complete set of instrument repair kits to set up their own instrument repair workshops. (Table 2).

**Table 2:** List of equipment in the instrument repair kit donated to participating hospitals.

No.	Equipment
1	3 oz. brass Hammer
2	Grinding dressing tool
3	Table vise
4	Bench grinder
5	Diamond file set
6	Diamond file set
7	Abrasive wheel
8	Electrican digital microscope
9	Center Punch
10	Work gloves
11	Sandpaper abrasive
12	Rotary tool kit
13	Polishing wheel
14	Resistance bands
15	Mini pilers

The main outcome for this phase was to create a pool of trainers that can go on to train others at their locations and regions.

#### Phase two

The goal of this phase was to step down the training and expand the pool of instrument repair technicians and the number of instruments repaired. Stepping down refers to the process of transferring knowledge and skills acquired during the phase 1 training to a broader group of participants, at a more localized level. Those trained in phase 1 are expected to disseminate the training to others, creating a cascading effect. It’s intended to build more capacity, ensure scalability without the need for centralized training, cut down cost of training as well facilitate sustainability.

The aim was to focus on the practical aspects of the training program using hands-on training and increase the number of instruments repaired and returned to use. The motivation for changing the mode of delivery for phase 2 was to enable the participants trained in phase 1 to pass on practical skills within their hospitals and nearby hospitals. Theoretical knowledge will be passed on in an informal but continuous mentoring manner as the trainers are working at the same location with the participants.

This phase consisted of only 1 week of hands-on training. It was determined from phase 1 that the 2-week hands-on period in stage 2 can be compressed to 1 week without compromising skills learnt. The participants in this phase did not go through the online theoretical component. The outcomes for this phase were increase in number of technicians with practical skills and increase in number of instruments repaired and returned to use.

This phase was conducted at 2 sites, the National Hospital, Abuja, Nigeria, and at the National Orthopedic Hospital, Enugu, Nigeria. These 2 locations were chosen for their ease of access in the northern and southern parts of the country. The participants were selected from Smile Train partner hospitals in each region. The participants in this phase were completely different from those trained in phase 1. The training was facilitated by three of the participants involved in phase one training.

### Ethics approval

Ethics approval was obtained from the Institutional Review Board (Health Research and Ethics Committee) of the National Hospital, Abuja, Nigeria, as part of the development and implementation of Nigeria’s NSOANP.

## Results

### Demographics and distribution of participants

A total of 36 hospital staff were trained. There were 24 (66.6%) participants in phase one and 12 (33.3%) in phase 2 (the 12 participants in phase 2 did not include any of the participants in phase 1). There were 20 males and 4 females in phase one and 8 males and 4 females in phase two (Table 3). All 24 participants in stage 1 of phase 1 passed the theoretical component with over 80% and proceeded to stage 2.

**Table 3:** Demographics of participants trained.

Demographics		Phase 1	Phase 2
Specialty	Perioperative nurses	12	5
	Biomedical technicians	8	7
Geopolitical zine	North East	4	
	North West	4	
	North Central	4	6
	South East	4	6
	South West	4	
	South South	4	

### Characteristics and types of instruments repaired

**Figure 1:**
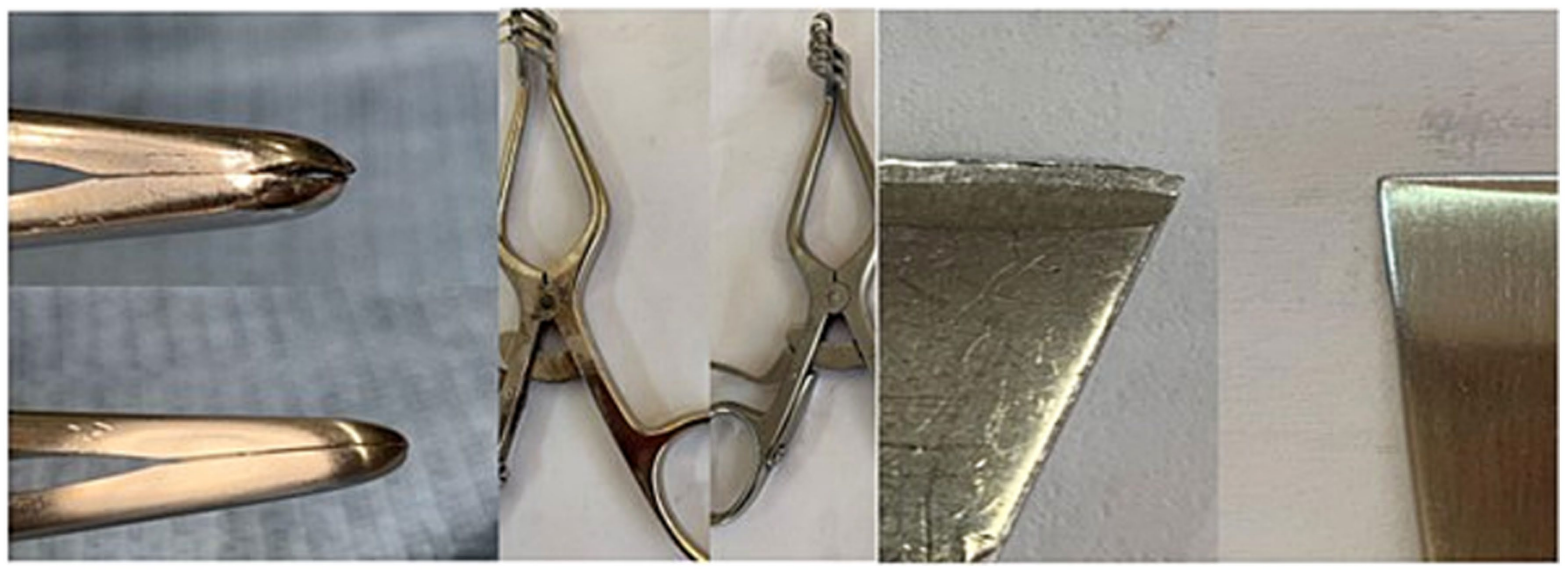
Instruments, before and after repair.

The details of specific instrument numbers repaired in phase one were not captured, as the main focus was on skills and knowledge acquisition. In phase two, 1,623 instruments were evaluated for repair. Seven (0.4%) instruments were determined to be completely unsafe for use, whereas 1,616 (99.6%) were repaired. The instruments considered refurbishable were repaired and returned for use (Figure 1).

The instruments used for repair were from general surgery, orthopedic surgery, plastic surgery, otorhinolaryngology, dental surgery, ophthalmology, obstetrics and gynecology. Overall, 81.8% of the instruments were from general surgery, 10% orthopedic surgery, 4.5% obstetrics and gynecology, 1.6% plastic surgery, 1.5% otolaryngology and dental surgery, and 0.5% ophthalmology. The most common set of instruments presented for repairs was different types of forceps (677, 41.9%), scissors (279, 17.3%), retractors (107, 6.6%), speculum (11, 0.05%) and others (542, 33.5%) consisting of a wide variety of instrument types.

Overall, the most common types of instrument repairs were buffing, polishing, rust removal and sharpening.

### Initial impact of training

#### Workforce impact

Among the 24 participants who completed the post-training survey in phase 1, 20 (83.6%) reported improved knowledge and confidence in the identification of instruments needing repair, whereas 4 (16.7%) still had difficulty identifying faulty instruments. Twenty-three (95.8%) affirmed that they had acquired adequate skills in the proper handling of surgical instruments and the repair of faulty instruments, whereas 19 (79.2%) affirmed that they could confidently carry out ongoing evaluation and maintenance of instruments in use. Only one (4.2%) participant felt ready to train others.

#### Institutional impact

**Figure 2:**
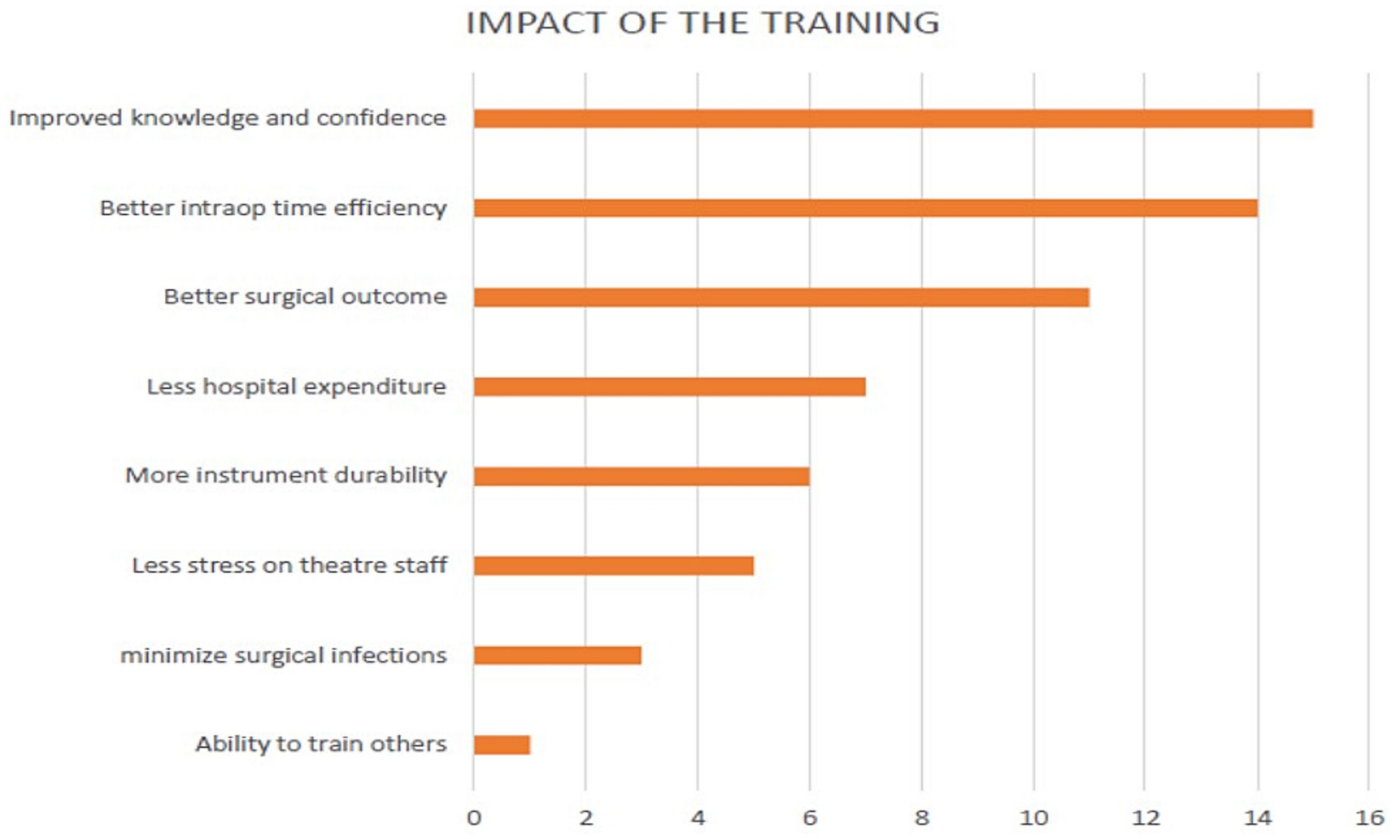
Participants perceived impact of SIRT training.

At the end of phase one, 14 (58.3%) participants agreed that the training improved operating time efficiency, whereas 11 (45.8%) agreed that it improved patient outcomes (Figure 2). Six (50%) of the 12 participating hospitals have established specific instrument repair work benches. Five (41.6%) participating hospitals have carried out in-hospital instrument repair drives to repair faulty instruments, and 2 (16.7%) have carried out surgical instrument repair drives for neighboring hospitals.

## Discussion

Faulty surgical instruments contribute significantly to mishaps during surgical procedures, with adverse implications for patient outcomes (5, 10). Poorly maintained instruments prolong the operating time, exposing patients to unnecessarily prolonged periods under anesthesia and its potential complications, especially in high-risk patients (11). Overall, this extends the operating time and reduces the efficiency of surgical processes and infrastructure management, limits access to surgical care and could negatively affect the cost-effectiveness of existing surgical systems (12). The impact on surgeons and other operating staff has not been well documented, but it is known to result in negative emotions during surgical procedures and disrupt workplace harmony among perioperative staff. This approach has the potential to increase the direct risk to the patient from the faulty instrument.

### SIRT training

In Nigeria, training in surgical instrument repair and maintenance is limited. This is compounded by limited resources for the purchase of new instruments to replace faulty instruments. This scenario worsens the scale of the problem with faulty instruments (10, 12). The situation is similar in many LMICs.

The cost of training in the maintenance of biomedical equipment, including surgical instruments, can reach as high as £2,250 per person (8). This often requires out-of-country travel, resulting in additional costs to institutions and countries with scarce resources. The immense benefits of locally contextualized instrument repair training delivered locally and internationally cannot be overemphasized. Our SIRT program had equitable geopolitical spread with the involvement of participants from the six geopolitical zones in the country. The provision of instrument repair training kits for participating institutions, resulting in the creation of institutional workbenches, has initiated a sustainable pathway. This is demonstrated by the ongoing repair of instruments at these hospitals and step down to other relevant personnel within the same hospital and at neighboring hospitals. The ultimate aim is to expand the pool of trained personnel and prospective trainers. The participants reported feedback emphasized improvements in confidence, knowledge and skills in instrument repair and maintenance.

### Challenges

A notable challenge of our SIRT training program is the initial cost of conducting in-country training. While this number was high, equipping participants to step down the training in their regions significantly reduces cost while creating and increasing the pool of repair technicians and trainers. Our model involved aligning the training with the priorities of a cleft charity, enabling partnership and funding to increase the impact of the work of the charity while building local capacity in surgical instrument repair and maintenance. This unique collaborative model has the potential to attract commitment from other funders as well as increased support from policy makers. The inclusion of training in health institution budgets would help to further ensure sustainability.

While the number of individuals currently trained is small, the capacity for expansion is immense. This approach urgently needs to be exploited to improve surgical safety in our setting and similar settings.

Although the participants expressed low confidence in their ability to train others, the initial training conducted by the participants was supervised by instructors. This helps improve the confidence of new trainers (previously trained participants) and enhances the quality of training. The lack of confidence of the prospective trainers to train others may well be because this was their very first in-depth experience with instrument repair and maintenance. In addition, they have not been involved in training others previously. This could be addressed in future trainings by incorporating a module on mentoring into the curriculum.

### Next steps

Formally incorporating a “training of trainers” module in the curriculum to address any currently existing gaps in step-down training would help improve the confidence of prospective trainers. This is being done as the expansion of the training program is planned for the future. The plan for scaling up is to deploy the 4-week approach (used in phase 1) to train the trainers and use the 1 week step down approach (used in phase 2) to expand the number of technicians with practical skills by passing on skills within each hospital and region.

Advocacy to potential funders leveraging their priorities is a key strategy that is currently being deployed. In addition, we also advise the inclusion of surgical instrument repair and maintenance in institutional budgets.

Objectively tracking the outputs of the program will help to determine the true impact of the training. For subsequent training, a baseline assessment of the areas of expected impact, such as impact on the workforce, patients, equipment and institutional processes, will be performed before training is deployed. This will help generate much needed objective evidence of impact to strengthen advocacy as well as drive expansion and progress.

### Recommendation

To sustain and expand the SIRT training program, it is crucial to formalize it within national health strategies, as well as incorporating surgical instrument maintenance into hospital budgets and institutional policies. Regular follow-up training, including a “train-the-trainer” model, enhances participants’ confidence and ensures wider dissemination of skills across more hospitals. Advocacy for additional funding from the government and international partners should be prioritized to support the program’s expansion. Furthermore, tracking the long-term impact on surgical outcomes and efficiency will provide essential data to strengthen future initiatives.

## Conclusion

The SIRT training program has made significant efforts to improve the maintenance and safety of surgical instruments across hospitals in Nigeria. By equipping biomedical technicians and operating room staff with essential skills and tools for repairing and maintaining surgical instruments, the program has addressed a critical gap in surgical safety. The initial success of the SIRT program highlights the value of local capacity-building initiatives in enhancing surgical healthcare delivery. However, scaling up this program and ensuring its sustainability will require continuous training, monitoring, and institutional support.

## Data Availability

The original contributions presented in the study are included in the article/supplementary material, further inquiries can be directed to the corresponding author.
